# Prevalence of sexually transmitted diseases in female athletes in São Paulo, Brazil

**DOI:** 10.1590/S1679-45082014AO2949

**Published:** 2014

**Authors:** Maíta Poli de Araujo, Henrique Truffa Kleine, Tathiana Rebizzi Parmigiano, Natalia Tavares Gomes, Graziela Pascom Caparroz, Ismael Dale Cotrim Guerreiro da Silva, Manoel João Batista Castello Girão, Marair Gracio Ferreira Sartori

**Affiliations:** 1 Universidade Federal de São Paulo, São Paulo, SP, Brazil

**Keywords:** Sports medicine, Sexually transmitted diseases, Vaginal smears, Papillomavirus infections, Real-time polymerase chain reaction

## Abstract

**Objective:**

To determine the prevalence of sexually transmitted diseases in female athletes.

**Methods:**

An observational, cross-sectional study was conducted including 50 female athletes with mean age of 20±3 years. Colposcopy, pap smear, and polymerase chain reaction for *Chlamydia trachomatis*, human papillomavirus and *Neisseria gonorrhoeae* were performed. Blood samples were collected to test for the human immunodeficiency virus, syphilis, hepatitis B and C. The athletes presenting clinical diseases or conditions identifiable by laboratory tests were treated and followed up in the unit.

**Results:**

Forty-six percent of the participants were unaware of sexually transmitted diseases. The prevalence of sexually transmitted diseases among athletes was 48% (24 cases). Human papillomavirus was the most frequent agent (44%). Considering the human papillomavirus genotypes, subtype 16 was the most prevalent (53%), followed by 11-6 (22%) and 18 (13%). Two athletes tested positive for *C. trachomatis*. There were no cases diagnosed of infection by *N. gonorrhoeae*, syphilis, hepatitis B, hepatitis C and human immunodeficiency virus. However, only 26 athletes had been vaccinated for hepatitis B.

**Conclusion:**

The prevalence of sexually transmitted diseases in female athletes was high. Primary prevention measures (hepatitis B and human papillomavirus vaccination) and secondary (serology, pap smears) must be offered to this specific group of women. The matter should be further approached in sports.

## RESUMO

**Objetivo:**

Determinar a prevalência de doenças sexualmente transmissíveis em mulheres atletas.

**Métodos:**

Estudo observacional, de corte transversal, que incluiu 50 mulheres atletas com idade média de 20±3 anos. Realizaram-se colposcopia, coleta de colpocitologia oncótica cérvico-vaginal e pesquisa para *Chlamydia trachomatis*, papilomavírus humano e *Neisseria gonorrhoeae*, pelo método do reação de cadeia de polimerase. Amostras de sangue foram obtidas para pesquisa de vírus da imunodeficiência humana, sífilis, hepatite B e C. As atletas que apresentaram doenças clínicas ou laboratorialmente identificáveis receberam tratamento e acompanhamento no serviço.

**Resultados:**

Dentre as participantes, 46% relataram desconhecimento acerca das doenças sexualmente transmissíveis. A frequência de doenças sexualmente transmissíveis nas atletas foi de 48% (24 casos). Isoladamente, o papilomavírus humano foi o agente mais frequente (44%). Considerando o tipo de genótipo do papilomavírus humano, o subtipo 16 foi o mais prevalente (53%), seguido do 6-11 (22%) e do 18 (13%). Duas atletas tiveram resultado positivo para *C. trachomatis*. Não foi diagnosticado nenhum caso de infecção por *N. gonorrhoeae*, sífilis, hepatite B, hepatite C e vírus da imunodeficiência humana. Contudo, somente 26 atletas haviam sido vacinadas para hepatite B.

**Conclusão:**

A prevalência de doenças sexualmente transmissíveis em mulheres atletas foi elevada. Medidas de prevenção primária (vacinação para hepatite B e papilomavírus humano) e secundária (sorologias e colpocitologia) devem ser fornecidas a esse grupo específico de mulheres. O assunto deve ser abordado no meio desportivo.

## INTRODUCTION

In 1991, basketball player Magic Johnson announced he had acquired immunodeficiency syndrome, commonly known as AIDS. The news had a major impact on the sports world, and many of his teammates began to get tested, because they were afraid of having acquired the disease.^(1)^ The truth is that up to this event, athletes were seen as super-heroes and little was known on the risk of acquiring sexually transmitted diseases (STD) while practicing sports and what was the prevalence of these conditions in athletes. The risk of contamination is currently known to be very low and the possibility lies on the presence of bleeding wounds.^(2,3)^

The risk of transmission of the human immunodeficiency virus (HIV) may occur in some contact sports, such as wrestling, *taekwondo*, box and American football. ^(2)^ However, the risk is extremely low, estimated to be around 1/85 million games.^(3,4)^ Conversely, transmission through contaminated syringes in athletes who use illicit drugs (doping), has been associated to several sports modalities.^(5)^

The risk of transmission of the hepatitis B virus is 50-fold greater than of the HIV virus. This occurs because the virus is resistant to drying, room temperature, to detergents and to alcohol, in addition to being stable on surfaces, during at least 7 days. Therefore, the transmission of hepatitis B in sports is estimated at about 1/10 thousand to 1/50 thousand games.^(2)^

Regarding hepatitis C, there have been reports on former football players that used injections of vitamin complexes.^(6-8)^

STD are among the five major causes for seeking medical care and the second major cause of not having a healthy lifestyle among women.^(9,10)^ Although most conditions are curable, many lesions increase the risk of HIV infection three-fold and others are precursors of neoplasms – such as human papillomavirus (HPV), *Chlamydia trachomatis* (CT) and *Neisseria gonorrhoeae* (NG) infections ^(7)^.

Some studies appointed a positive association between being an athlete and having risky sexual behavior.^(11,12)^ Young athletes begin contact with the opposite sex early, on competition trips, and the lack of knowledge on transmission of STD makes many of them not practice safe sex.^(13)^ Moreover, this population has a greater consumption of anabolic substances and a higher number of sexual partners.^(14-16)^

High performance athletes and, especially those in team sports, have little perception of risks, and also engage in non-programmed sexual activity.^(17)^ In soccer, over 80% of players declare acknowledging HIV but few (20%) know about other STD.^(18)^ They also have questions on how the virus is transmitted, given many believe in contamination by exchanging shirts and kissing.^(18)^

In recent years, professionals who work with athletes have proposed STD screening at the beginning of every season, using a pre-participation sports assessment (PPSA).^(19)^ It would be a timely moment for detecting these diseases, treating them whenever necessary, and counseling on means of prevention.

## OBJECTIVE

To determine the prevalence of sexually transmitted diseases among women athletes

## METHODS

An observational, cross-sectional study was performed at the Sports Gynecology Sector of the *Escola Paulista de Medicina da Universidade Federal de São Paulo* (UNIFESP), from January to July 2011. The Project was approved by the Ethics Committee of the institution, under number 1,269/08. All participants read and signed the consent form.

Investigators went to the athletes' training site and explained the objective of the study. Those who agreed to participate answered a questionnaire that emphasized gynecological data (age at menarche, characteristics of the menstrual cycle, contraceptive methods, past sexual and obstetric history and history of STD). Athletes, family members and coaches were offered talks on STD/AIDS on the training site and, after answering questions, athletes were invited to come to the outpatient clinic, where they would be seen by a gynecologist.

Women who practiced performance sports in Olympic modalities of the International Olympic Committee (IOC) were considered athletes. All of them had been federation affiliates for at least three years and had a mean training load of 20 hours/week.

At the outpatient clinic, blood samples (to diagnose syphilis, HIV, hepatitis B and hepatitis C) were drawn, and cervical and vaginal secretion were collected during colposcopy (for pap smear, CT, NG and HPV tests). A biopsy of the lower genital tract was done whenever necessary.

The flocculation method was used (VDRL), along with the titer for syphilis; whenever necessary the result was confirmed by passive hemagglutination methods (HTPA) and EIE (ELISA).

HIV was diagnosed by two ELISA tests. If any of the two were positive, immunofluorescence tests were performed. If a question remained a Western blot test was performed.

ELISA was used for hepatitis B serology, using the following the algorithm: determination of titers of HBs antigen and total anti-HBc antibodies. If both were negative, the result was considered negative. If the HBsAg was negative and total Anti-HBc positive, an anti-HBs antibody detection test was performed, and the positive result indicated immunity or previous hepatitis B infection.

Serology for hepatitis C used two techniques: EIA-Hepanostika^®^ qualitative immunoenzyme test and MONOLISA™ anti-HCV plus, an indirect immunoenzyme test.

Regarding cervical and vaginal samples, results with superficial, intermediate squamous cells and/or endocervical cells without abnormalities were considered normal cytology, as were those with squamous metaplasia and/or with inflammatory changes. A diagnosis of low grade lesion, high grade lesion, squamous carcinoma, squamous cell atypia of undetermined significance and glandular atypia of undetermined significance were considered abnormal cytology.

Real time polymerase chain reaction (PCR) was used to test for CT, NG and HPV. All athletes with clinical or identifiable laboratory diseases were treated and followed up at the service.

The population was analyzed as to age, race and sports modality. The following gynecological data were analyzed: age at menarche, menstrual cycles, contraceptive method, and number of pregnancies and of deliveries. Analysis of variables was descriptive. Minimum and maximum values, medians, means and standard deviations were used for quantitative variables.

## RESULTS

Fifty athletes with a mean age of 20±3 years were assessed. Of the total, 66% declared themselves as white and 34% as non-white. As to socioeconomic status, 35% of participants had completed College, 11% High School and 4% Elementary School. Monthly family income ranged between R$ 370.00 and R$ 2,100.00.

Table 1 shows the characteristics of the sample as to sports modality and to choice of contraceptive method. Most participants (40%) took part in athletics and martial arts (36%) teams. Regarding contraception, 29 athletes used oral hormone contraceptives, 12 athletes used male condoms and 6 athletes did not use any method. As to gynecological data, the mean age of menarche was 13±1 years, with a minimum of 9 and maximum of 15 years. Regular menstrual cycles were reported by 82% of athletes and 18% had irregular menstrual cycles. Regarding parity, 90% were nullipara.

**Table 1:** Distribution of sports modality and contraceptive methods

Variables		n (%)
Sports modality		
	Athletics	20 (40)
	Martial arts	18 (36)
	Basketball	1 (2)
	Dance	3 (6)
	Indoor soccer	2 (4)
	Handball	4 (8)
	Sychonized swimming	1 (2)
	Swimming	1 (2)
Contraceptive method		
	Oral hormone contraceptive	29 (58)
	Male condom	12 (24)
	Female condom	1 (2)
	Monthly injectable	1 (2)
	IUD	1 (2)
	No method	6 (12)

IUD: progesterone releasing intrauterine system.

When each athlete was asked on knowledge of STD, 46% declared not having knowledge on such conditions, and five athletes reported a previous HPV infection.

The prevalence of STD among athletes analyzed was 48% (24 cases). HPV was the most frequent agent (44%) and, considering mixed infections, there was a case of CT com HPV. No cases of infection by NG, or syphilis, hepatitis B, hepatitis C and HIV were diagnosed (Table 2).

**Table 2:** Prevalence of sexually transmitted diseases, alone or associated

STD	n (%)
HPV	22 (44)
Chlamydia	1 (2)
Gonorrhoeae	0 (0)
Syphilis	0 (0)
HIV	0 (0)
Hepatitis B	0 (0)
Hepatitis C	0 (0)
HPV+Chlamydia	1 (2)

STD: sexually transmitted diseases; HPV: human papillomavirus; HIV: human immunodeficiency virus.

Regarding the type of HPV genotype, that was detected in 23 of 50 women athletes, subtype 16 was the most prevalent (53%), followed by 6-11 (22%) and by 18 (13%) (Table 3).

**Table 3:** Type of human papillomavirus genotype

HPV type	n (%)
16	12 (53)
18	3 (13)
6-11	5 (22)
53	1 (3)
66*	2 (9)

* One of the athletes with HPV genotype 66 also had a positive result for chlamydia. HPV: human papillomavirus.

Although no cases of hepatitis B were detected, 24 (48%) athletes were not immune to hepatitis B virus (negative anti-HBs).

Three athletes presented low grade cervical lesions and one athlete had a high grade lesion detected by the cervical-vaginal cytology.

## DISCUSSION

Although muscle-skeletal lesions are strongly associated with exercising, infectious diseases cause significant morbidity among athletes.^(20)^ The results of the present study alert on the importance of measures to explain and orient about STD in sports, given half of athletes had no knowledge on these conditions.

The sports environment has a distorted image of being healthy and with no risk behaviors. Several authors appointed toward an excessive use of alcohol, tobacco, illicit drugs and unprotected sex among young athletes.^(21)^ Moreover, premature initiation of sex life and of non-programmed intercourse contribute to the growth in susceptibility to STD infections and to unwanted pregnancy.^(12,22)^

The general prevalence of STD among athletes was high, and the isolated or associated occurrence with HPV should be underscored. The prevalence of the HPV genotype with high risk for cervical cancer is also worth mentioning.^(16,18)^

The prevalence of HPV among asymptomatic women seen at public gynecological clinics, using the PCR method, is about 16% – much lower than the values found among athletes.^(23)^ A possible explanation would be the complex interaction between the immune system and exercise. In general, moderate intensity exercise promotes protection against infections, because it drives cell immune response (mainly of type 1 T-*helper* – Th1 cells). However, high intensity activities generate increase in the concentrations of anti-inflammatory cytokines (type 2 T-*helper* – Th2), increasing susceptibility to infections.^(24)^

The higher prevalence of oncogenic HPVs among athletes was a concerning finding. Epidemiological studies showed that HPV 16 is responsible for most cervical neoplasms, followed by HPV 18. HPVs 6 and 11 account for 90% of genital warts. Although infection by oncogenic HPV is a necessary condition to develop cervical cancer, it is insufficient. In two years, the infection reverts spontaneously in almost 90% of cases. Moreover, the progression of infection to cervical cancer is very slow and controllable. Thus, cervical cancer screening, using pap smears, should be part of the routine for young athletes, along with other routine tests requested regularly by sports physicians.

HPV vaccination is part of the recommendations proposed by the Brazilian Federation of Gynecology and Obstetrics Societies (FEBRASGO) and by the Brazilian Association of Immunizations (SBIm) and should be better promoted among athletes. A timely moment to offer vaccination would be PPSA.

The United States and Australia have put in place routine tests for CT and gonorrhea during the PPSA.^(25)^ In one of the studies with 9 to 12-year-old boys and girls, the prevalence of CT in girls was 6.5% and of gonorrhea 2.0%.^(26)^ In another study, the general prevalence of CT among athletes was 2.7% (3.2% men and 2.2% women).^(27)^ In our study, the prevalence of CT was in agreement with the literature, highlighting that one of the athletes had a co-infection with the HPV virus.

CT infections have a high morbidity and also have a preference for youth between 15 and 29 years. Considered as having a silent epidemiology, its diagnosis is critical, given the infection is asymptomatic in almost 80% of cases. One of the problems of persistent infection by *Chlamydia* is its cervical carcinogenesis capacity, which occurs through proteins synthetized by bacteria.^(28)^ These proteins have an anti-apoptotic action during persistent infection, which eases the action of oncoproteins on cells simultaneously infected by high risk HPV (HPV types 16 and 18).^(28)^ Another severe sequela is pelvic inflammatory disease which can lead to ectopic pregnancy and to infertility.^(29)^

There is a consensus that the major means of transmission of STD among athletes is not while practicing sports, but similar to the general population – by unprotected sex and exchange of contaminated syringes.^(30)^ Therefore, the prevention of these infections is still one of the most efficacious and less costly measures that exist.^(31)^

The major international organizations for athletes have stimulated hepatitis B vaccination, contact sports judges wearing gloves, the interruption of a game when there is a bleeding lesion and the education of coaches, fitness coaches and athletes on STD/AIDS.

Athletes should be asked about their knowledge and difficulties regarding sexuality and prevention of STD, which would also mean helping them to deal with their own vulnerability.

As the sports world is a strong information media, capable of spreading several topics to the general population, athletes with appropriate knowledge of STD may boost the importance of using preventive measures, such as safe sex and vaccination for some diseases.

## CONCLUSIONS

The prevalence of sexually transmitted diseases in female athletes analyzed was high and the human papillomavirus was the most frequent agent. Results point toward the vulnerability of athletes to sexually transmitted diseases and toward the need for primary (hepatitis B and human papillomavirus vaccination) and secondary (serology, colposcopy and cytology) prevention measures in this specific group of women.
